# Supplementary material for: Development of a computer-aided design software for the quantitative evaluation of aesthetic damage
Source: PLoS One. 2019 Dec 18;14(12):e0226322. doi: 10.1371/journal.pone.0226322 (PMC6919621; doi:10.1371/journal.pone.0226322)
Supplement: S1 Code site — 1. (ZIP) [file pone.0226322.s001.zip › [EESC_jr][Projeto]Relatorio_07_21/reports/9/2017.02.04/14:37:14/report.pdf]

Preâmbulo

Na data de 01/01/2017 no horário 10:09, o Perito(a) Sr. Rafael Yonezawa de Mello, Estudante, designado(a)para proceder ao exame pericial solicitado em: Carolina Caetano Anraki, do gênero Feminino, nascido(a) em 06/05/1997, ocupação Estudante, portador(a) do RG 1231312414 SSP-SP, cujo endereço Avenida Henriqueta Soares, 51, passa a expor a avaliação de dano estético corporal.

Exame objetivo da lesão

A lesão ora objeto de análise teve como natureza Erro profissional ocorrido em 31/12/2016. O prejuízo estético consiste em lesão cicatricial consolidada na região: Tórax no lado esquerdo.

Trata-se de cicatriz Quelóide de tamanho/frequência Múltipla: Puntiforme de orientação Vertical de pigmentação Hipocrômica medindo 1 centímetro(s) na horizontal, 10 centímetros na vertical e 5 milímetros de Altura. De contornos Regulares. Perceptivel em 15 centímetros de distância nas relações íntimas. Perceptivel em 1 metros nas relações sociais. A lesão tem repercução na vida diária afetiva, laboral conforme fotos anteriores e atuais..

Conclui-se que classificada segundo os parâmetros de dano estético AIPE - Análise do Impacto do Prejuízo Estético (escala de seis níveis e pontuação entre 0 e 50 pontos), como sendo dano estético relativo à área 1, a lesão é caracterizada por 13 pontos e grau de prejuízo estético: Leve.

Escore final: 13;
Grau de prejuízo: Leve.

São Carlos, 01/01/2017

____

Sr. Rafael Yonezawa de Mello
